# Suppression of breast cancer metastasis through the inactivation of ADP-ribosylation factor 1

**DOI:** 10.18632/oncotarget.11185

**Published:** 2016-08-10

**Authors:** Xiayang Xie, Shou-Ching Tang, Yafei Cai, Wenhu Pi, Libin Deng, Guangyu Wu, Alain Chavanieu, Yong Teng

**Affiliations:** ^1^ Department of Oral Biology, Augusta University, Augusta, GA, USA; ^2^ Department of Pediatrics, Emory Children's Center, Emory University, Atlanta, GA, USA; ^3^ Georgia Cancer Center, Augusta University, Augusta, GA, USA; ^4^ Tianjin Medical University Cancer Institute and Hospital, Tianjin, P.R. China; ^5^ Department of Pharmacology and Toxicology, Augusta University, Augusta, GA, USA; ^6^ Institut des Biomolécules Max Mousseron (IBMM), UMR 5247, Université de Montpellier, CNRS, ENSCM, France; ^7^ Department of Biochemistry and Molecular Biology, Augusta University, Augusta, GA, USA

**Keywords:** ARF1, breast cancer, metastasis, LM11, zebrafish

## Abstract

Metastasis is the major cause of cancer-related death in breast cancer patients, which is controlled by specific sets of genes. Targeting these genes may provide a means to delay cancer progression and allow local treatment to be more effective. We report for the first time that *ADP-ribosylation factor 1* (*ARF1*) is the most amplified gene in *ARF* gene family in breast cancer, and high-level amplification of *ARF1* is associated with increased mRNA expression and poor outcomes of patients with breast cancer. Knockdown of *ARF1* leads to significant suppression of migration and invasion in breast cancer cells. Using the orthotopic xenograft model in NSG mice, we demonstrate that loss of *ARF1* expression in breast cancer cells inhibits pulmonary metastasis. The zebrafish-metastasis model confirms that the *ARF1* gene depletion suppresses breast cancer cells to metastatic disseminate throughout fish body, indicating that ARF1 is a very compelling target to limit metastasis. ARF1 function largely dependents on its activation and LM11, a cell-active inhibitor that specifically inhibits ARF1 activation through targeting the ARF1-GDP/ARNO complex at the Golgi, significantly impairs metastatic capability of breast cancer cell in zebrafish. These findings underline the importance of ARF1 in promoting metastasis and suggest that LM11 that inhibits ARF1 activation may represent a potential therapeutic approach to prevent or treat breast cancer metastasis.

## INTRODUCTION

Death due to breast cancer results largely from metastatic spread of the disease [1, 2]. This process encourages cells to break apart from the tumor and travel through the body to another organ [2, 3]. It has become increasingly clear that metastatic progression correlates with the deregulation of certain gene sets in the primary tumor [4, 5]. Therefore, the identification of key molecules that control metastatic signaling cascades holds the best opportunity to design new therapeutic strategies for advanced breast cancer.

The human *ADP-ribosylation factor (ARF)* gene family has 5 members (*ARF1*, *ARF3*, *ARF4/ARF2*, *ARF5* and *ARF6*), which encode 5 ARF proteins categorized as class I (ARF1 and ARF3), class II (ARF4 and ARF5) and class III (ARF6) [6–8]. Like the Ras superfamily of proteins, ARFs are small GTPases and their functions are highly regulated by switching between active GTP- bound and inactive GDP-bound conformations [9, 10]. ARF1 and ARF6 are well characterized as crucial regulators for vesicular trafficking, and their roles have been implicated in the cancer progression. ARF6 is often overexpressed in many types of cancer and facilitates epithelial-mesenchymal transition and invasiveness [11, 12]. ARF6 anchors to the plasma membrane, where it recruits ARNO and coordinates membrane trafficking and cytoskeleton remodeling [13]. Unlike ARF6, ARF1 especially regulates translocation of proteins from trans-Golgi network to plasma membrane, and directly activates signaling molecules [7, 14–16].

In prostate cancer, we have linked ARF1 to the hyperactivation of mitogen activated protein kinase (MAPK) Raf1/MEK/ERK1/2 pathway [7]. In breast cancer, ARF1 has been reported to be involved in promoting cell proliferation and migration via multiple well-known signaling cascades. For example, ARF1 controls cell adhesion by regulating the recruitment of key focal adhesion proteins (such as paxillin, talin and FAK) to β1-integrin [17], controls cell proliferation by regulating pRB/E2F1 activity and gene expression [15], and controls cell migration and invasion by regulating the activation of Rac1 and PI3K/AKT pathways [14, 16]. Most recently, ARF1 is shown to act as a molecular switch to activate EGF-mediated responses and mediate the sensitivity of triple negative breast cancer cells to EGFR tyrosine kinase inhibitors [18]. On the basis of these clues, we wondered whether ARF1 functions as a metastasis promoter in breast cancer to drive metastasis. Here we provide evidence for such a role, and demonstrate that ARF1 is a very compelling target to limit breast cancer metastasis. Inactivating ARF1 may have a potential therapeutic value in this regard.

## RESULTS

### High-level amplification of *ARF1* gene in breast cancer

**Figure 1:**
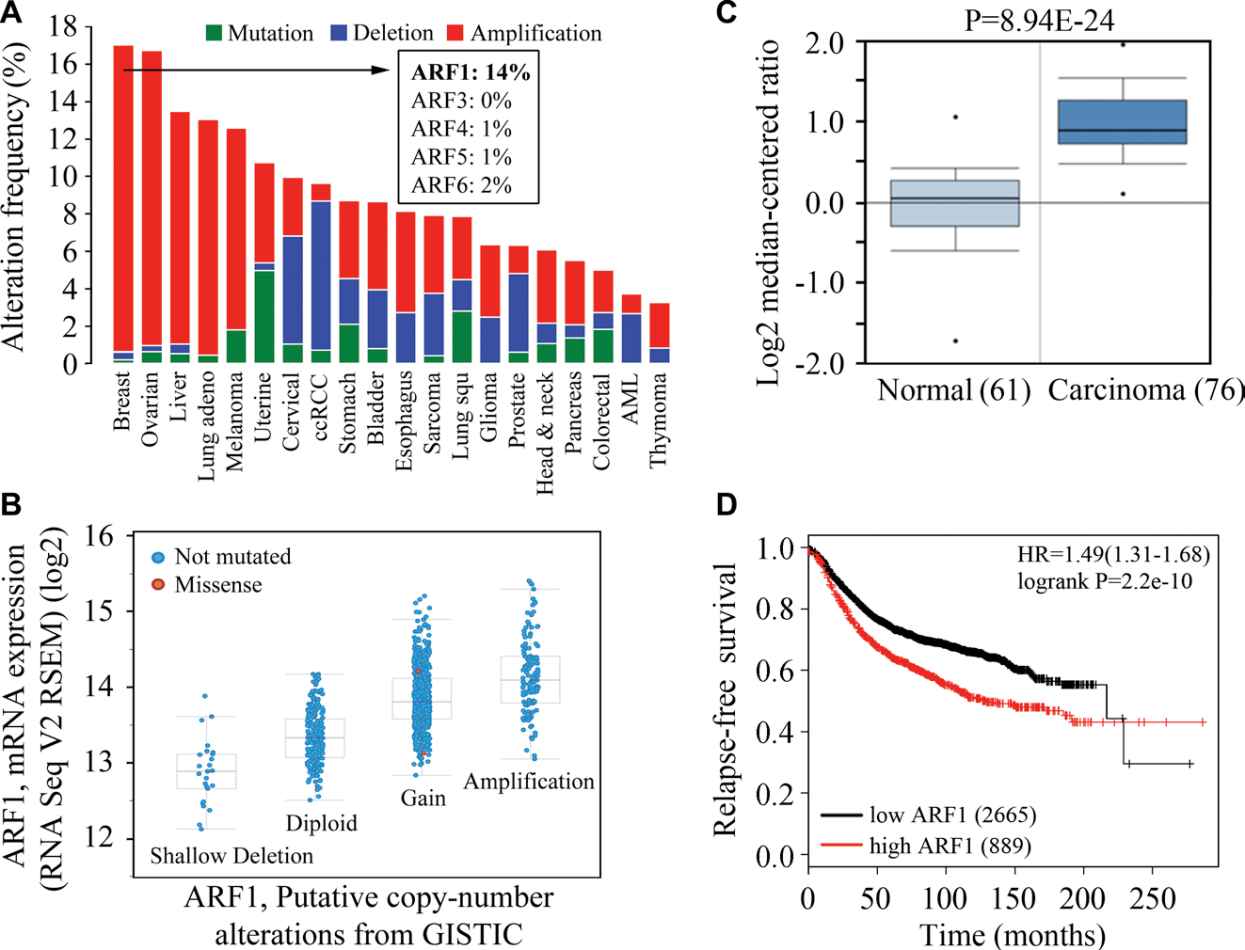
High-level amplification of *ARF1* is associated with increased mRNA expression and poor outcomes of patients with breast cancer (**A**) Summary graph of genetic alterations of the *ARF* genes in individual studies deposited in the cBioPortal. The amplification frequency of *ARFs* in breast cancer is shown in the inset. (**B**) A plot of the correlation between copy number alterations and mRNA expression of the *ARF1* gene. (**C**) Analysis of *ARF1* expression in breast normal and cancer tissues using Oncomine database. (**D**) Kaplan-Meier plot of RFS shown for breast cancer patients with high (red) and low (black) expression levels of the *ARF1* gene.

To explore whether the *ARF* genes contribute to the occurrence and development of cancer, we investigated the genetic alteration of a panel of the *ARF* gene family using publicly accessible TCGA datasets (see Materials and methods). Intriguingly, amplification of the *ARF genes* was found in 17% of cases of breast cancer, which was the highest in all the examined cancer types (Figure 1A). Amplification was the predominant type of alteration for *ARF1* gene and its frequency was much higher (14% of cases) than other family members in breast cancer. Functional plotting of the corresponding mRNA level in relation to genetic status of *ARF1* revealed that amplification of *ARF1* was associated with increased mRNA expression (Figure 1B). To further validate these findings, the relative expression of the *ARF1* transcript was examined in breast cancer entities from the Oncomine database, which showed *ARF1* expression levels were significantly higher in cancer than normal tissues (Figure 1C). Univariate survival analysis (Kaplan-Meier method and log-rank test) revealed that breast cancer patients with low levels of *ARF1* expression significantly improved relapse-free survival as compared with high expression levels (Figure 1D), which is likely to be related to its involvement in the lethal and advanced forms of breast cancer.

### ARF1 is upregulated in human breast cancer tissues

**Figure 2:**
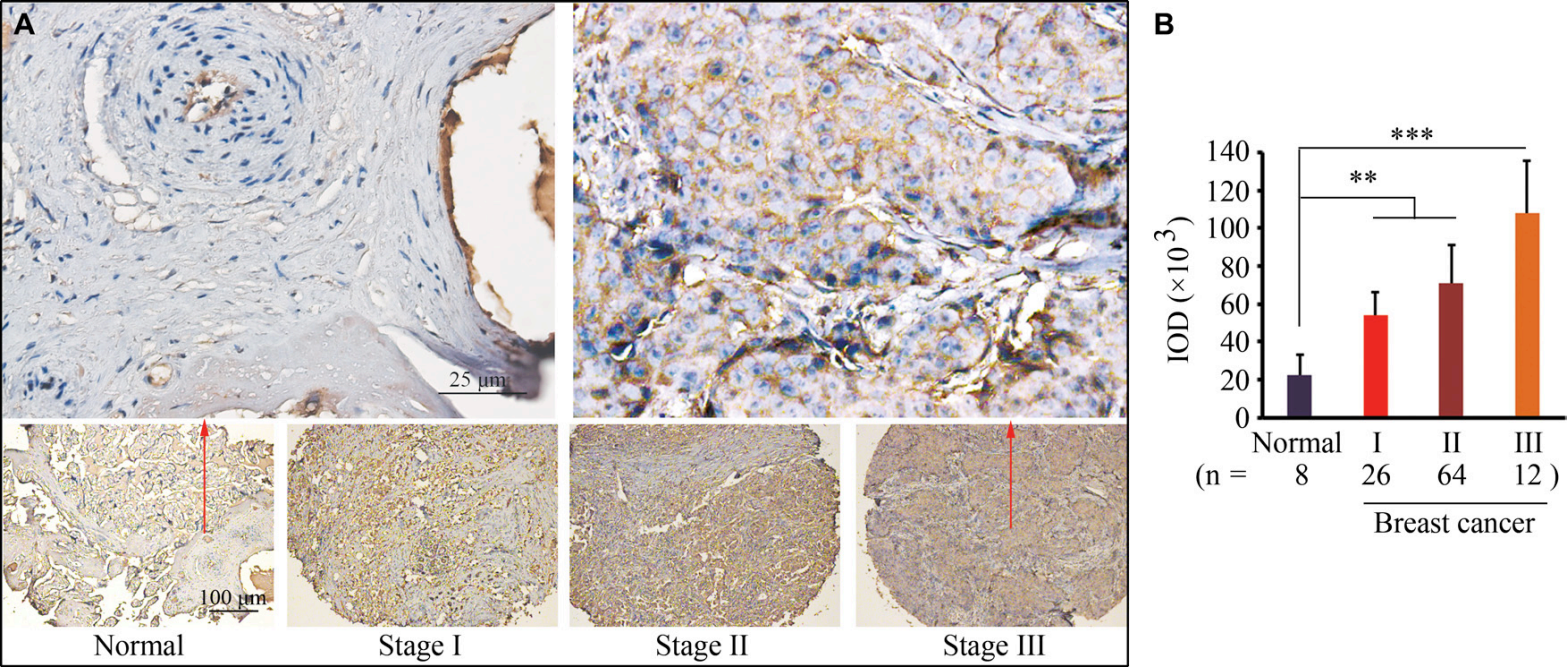
ARF1 is upregulated in human breast cancer tissues (**A**) Representative IHC results for ARF1 expression in breast cancer tissue arrays. (**B**) Quantitative data of staining intensity presented as integrated optical density (IDO). **p < 0.01; ***p < 0.001.

To validate the ARF1 expression pattern at protein levels, breast cancer tissue microarrays were used for immunohistochemistry (IHC) analysis. Our data indicate remarkably increased levels of ARF1 in primary breast cancer tissues compared with normal breast epithelium, and strong membrane staining of ARF1 in advanced breast cancer (Figure 2A). Most interestingly, higher levels of ARF1 were associated with higher cancer stages (Figure 2), supporting its critical role in breast cancer progression.

### Loss of *ARF1* expression suppresses metastasis in breast cancer

**Figure 3:**
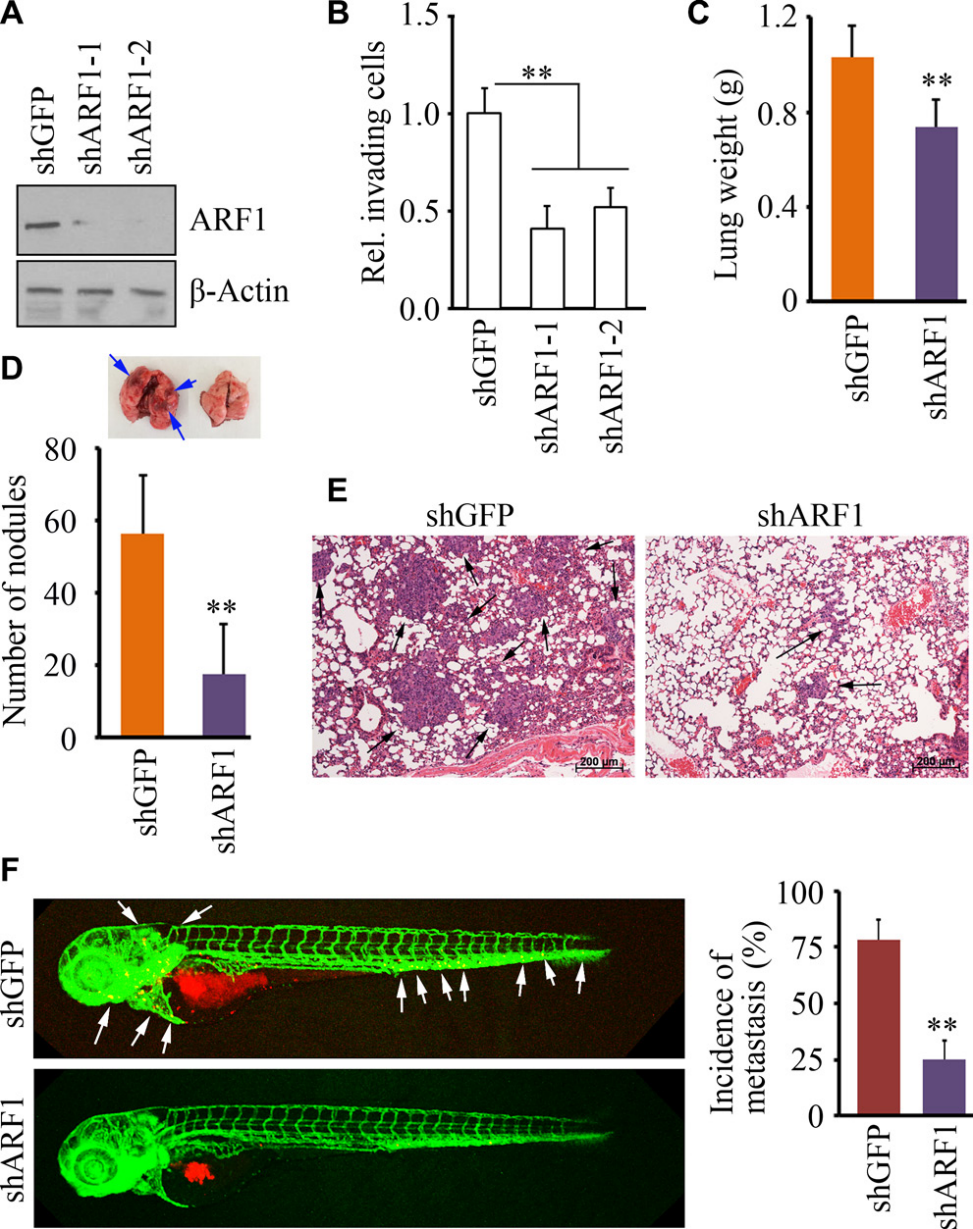
Knockdown of ARF1 leads to reduced cell invasion and metastasis in breast cancer (**A**) The effect of shRNA-mediated *ARF1* knockdown on MDA-MB-231 cells. (**B**) The effect of *ARF1* depletion on cell invasion. (**C, D, E, F**) The effect of *ARF1* depletion by shRNA on pulmonary metastases in the orthotopic mice model of breast cancer. (C, D) Quantitative data of lung weigh and gross surface pulmonary metastases. (E) Representative H&E stained lung sections from tumor-bearing mice. Six weeks after injection with MDA-MB-231 cells, the lungs from the NSG mice sacrificed were excised for pathological and histological analysis. Black arrows indicate representative metastatic foci. (F) The effect of *ARF1* depletion by shRNA on metastatic dissemination in zebrafish. White arrows indicate disseminated MDA-MB-231 cells in the fish body. Quantitative data are shown in right panel. **p < 0.01.

To better understand the role of ARF1 in breast cancer, we used shRNA constructs to inhibit *ARF1* expression in high-invasive breast cancer MDA-MB-231 cells (Figure 3A). Using two different shRNA constructs, knockdown of *ARF1* led to significantly reduced potential in cell invasion within 24 hours (Figure 3B) with modest decreased cell proliferation. To explore the importance of *ARF1* in metastasis *in vivo*, MDA- MB- 231 cells with *ARF1* knockdown were injected into the mammary fat pad of NSG mice and metastasis was monitored in these orthotopic breast cancer models. When pulmonary metastasis was examined at the conclusion of the experiment, mice injected with the knockdown control cells showed more nodules on the lung surface with heavier weights (Figure 3C and 3D), compared with those injected with *ARF1* knockdown cells. Histological analysis of these lungs further revealed a notable decrease in the number and size of metastatic foci on lung section when *ARF1* was depleted (Figure 3E).

Owing to the transparent and immunoprivileged nature of zebrafish embryos, we have recently established a zebrafish-metastasis model through a critical evaluation of various types of human cancer cells [19–21]. To determine whether the phenotype results from *ARF1* knockdown is reproducible in the zebrafish-metastasis model, we generated tumor-bearing zebrafish through injecting *ARF1* knockdown MDA-MB-231 cells and the knockdown control cells. Consistent with the findings from NSG mice, metastatic dissemination of the knockdown control cells was seen in 76% zebrafish at 2 days post-injection (dpi), whereas *ARF1* knockdown cells were only disseminated in 24% of zebrafish (Figure 3F). These observations demonstrate that *ARF1* is required for breast cancer progression and acts as a metastasis promoter.

### LM11 inhibits ARF1 activation in breast cancer cells in a dose-dependent manner

**Figure 4:**
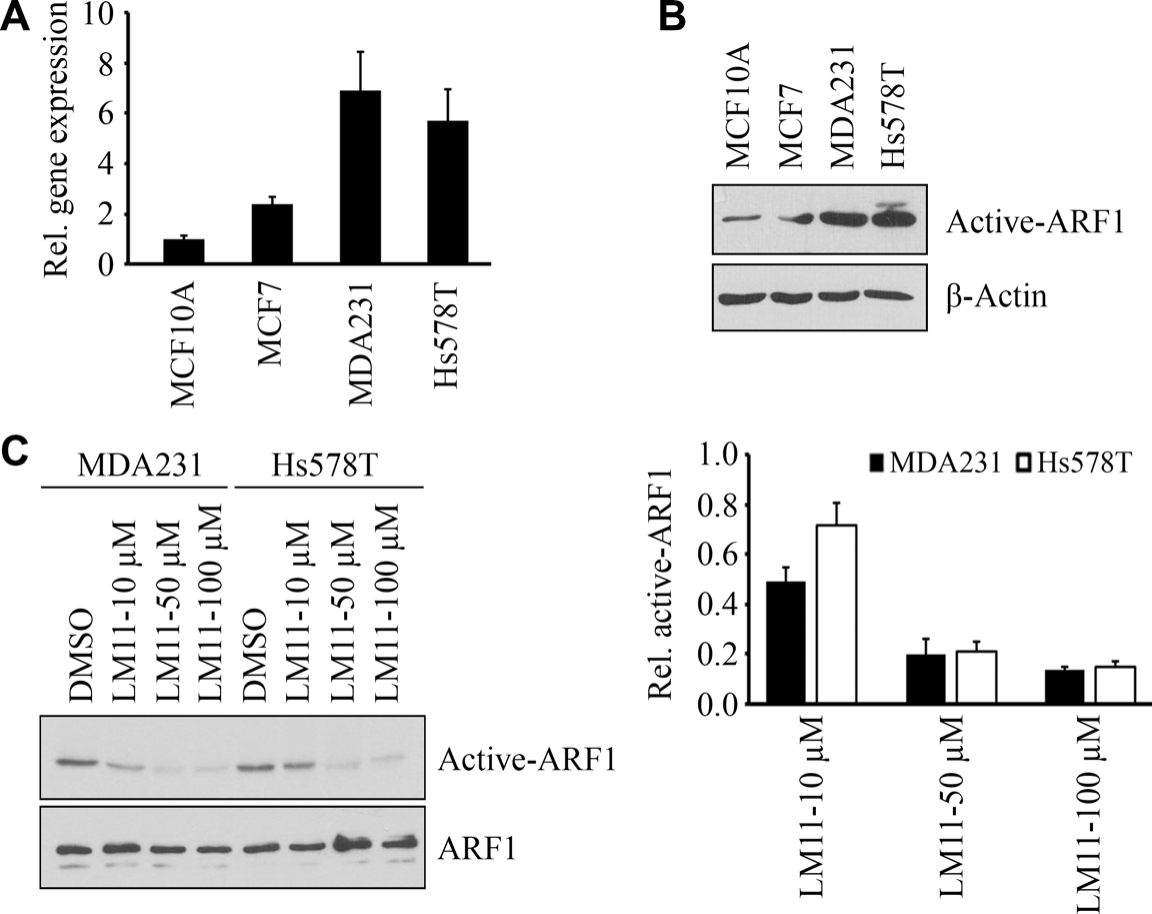
LM11 inhibits ARF1 activation in breast cancer cells (**A**) The relative expression levels of *ARF1* in breast normal and cancer cells. (**B**) The activation of ARF1 in breast normal and cancer cells. (**C**) The effect of LM11 on the activation of ARF1 in breast cancer. Quantitative data of relative ARF1 activation (LM11 *vs* DMSO) are shown in right panel.

We next determined levels of ARF1 expression and activation status in well-established human breast cancer cell lines. Real-time RT-PCR revealed elevated expression levels of *ARF1* in cancer cells (MCF7, MDA-MB-231 and Hs578T) when compared to human MCF10A mammary epithelial cells (Figure 4A). Moreover, the expression levels of ARF1 in two triple-negative cell lines (MDA-MB-231 and Hs578T) were much higher than those in MCF7 cells (Figure 4A). Interestingly, the levels of active GTP-bound ARF1 were strongly correlated with ARF1 expression in breast cancer cells (Figure 4B).

The data shown above suggest that inhibiting ARF1 expression may be a means of suppressing aggressive stage of breast cancer. However, there are no drugs directly targeting ARF1 expression. ARF1 is a small GTPase and its function largely dependents on the active form [7, 9, 10], therefore, we investigated the efficacy of the inhibitors blocking ARF1 activation. We have previously reported that a small molecule LM11 can effectively impair ARF1 activation in HeLa cells through targeting ARF1-GDP and ARF nucleotide-binding site opener (ARNO) in regions close to the ARF1/ARNO interface [22, 23]. As shown in Figure 4C, LM11 also inhibited ARF1 activation in breast cancer cells, and this effect was dose dependent.

### LM11 inhibits cell viability and invasion in breast cancer cells

**Figure 5:**
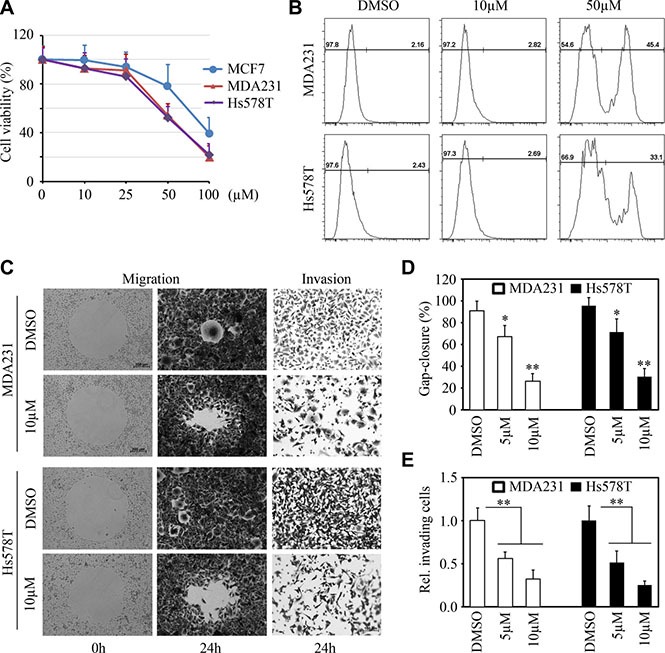
LM11 exhibits potent *in vitro* cytotoxicity and inhibits migration and invasion of breast cancer cells (**A, B**) The effect of LM11 on cell viability in breast cancer cells. MCF7, MDA-MB-231 and Hs578T breast cancer cell lines were treated with different doses of LM11 (10–100 μM) for 24 hours and cell viability was determined by CellTiter-Glo^®^ Luminescent cell viability kit (A). LM11-treated MDA-MB-231 and Hs578T cells were stained with Zombie Aqua^TM^ dye and cell viability was determined by flow cytometry (B). (**C, D, E**) The effect of LM11 on cell migration and invasion in breast cancer cells. Representative images of these assays shown in (C) and quantitative data shown in (D) and (E). MDA-MB-231 and Hs578T cells were treated with LM11 for 24 hours and cell migration and invasion were determined by Gap closure and Boyden chamber, respectively. *p < 0.05; **p < 0.01.

To specifically evaluate the effect of LM11 on breast cancer, we determined the *in vitro* cytotoxicity of LM11 in three breast cancer cell lines. Cell viability assays using CellTiter-Glo^®^ Luminescent cell viability kit (Promega, Madison, MI) showed that the IC_50_ (50% inhibitory concentration) of LM11 in the examined breast cancer cell lines ranged from 40 μM (MDA-MB-231 and Hs578T) to 75 μM (MCF7) (Figure 5A). Flow cytometry-based analysis further indicated, unlike high dose (50 μM), low dose of LM11 (10 μM) only modestly affected cell viability of breast cancer cells (Figure 5B). We thus determined LM11 effect on cell motility at low dose. Gap closure migration assays showed that LM11 significantly inhibited cell migration in both MDA-MB-231 and Hs578T cells (Figure 5C and 5D). Moreover, transwell invasion assays demonstrated that LM11 treatment led to a sharp decreased in invasion potential in these cells (Figure 5C and 5E). These data indicate that LM11 exhibits potent *in vitro* cytotoxicity and suppression of invasion in breast cancer cells.

### LM11 effectively suppresses breast cancer metastasis

**Figure 6:**
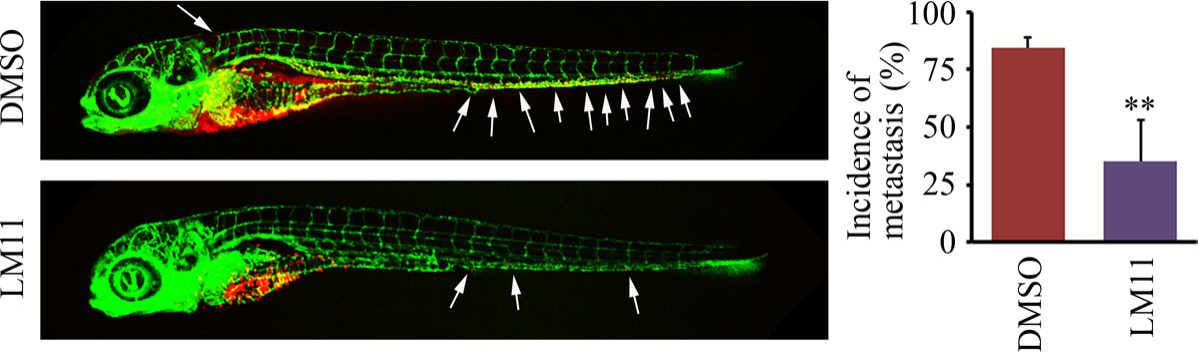
LM11 effectively suppresses breast cancer metastasis in the zebrafish model Tumor-bearing zebrafish were treated with 1 μM of LM11 for 4 days, and LM11 efficacy in metastatic dissemination was determined by confocal microscopy. White arrows indicate disseminated MDA-MB-231 cells in the fish body. Quantitative data are shown in right panel. **p < 0.01.

The zebrafish-metastasis model can evaluate the metastatic ability of cancer cells [19–21, 24]. We have shown above (Figure 3) that zebrafish robustly reported the decreased metastasis potential in breast cancer cells where the *ARF1* gene was knocked down. Therefore, we used this model to evaluate the efficacy of LM11 in suppression of breast cancer metastasis. To ensure that the effect was specific to tumors and did not affect the zebrafish development and growth, the overall length of each fish was recorded under bright field illumination before and after LM11 treatment. At the concentration ranging from 0.1–1 μM, LM11 did not alter overall fish growth within 1 week of treatment, as compared with the treatment of vehicle (DMSO) (data not shown). Most importantly, 1 μM of LM11 significantly inhibited MDA-MB-231 cells to disseminate from the perivitelline cavity to fish body (Figure 6). These observations demonstrate that LM11 has strong anti-cancer activities through suppressing the phenotypes associated with breast cancer metastasis.

## DISCUSSION

ARFs are a family of GTPases involved in a range of cellular functions, including critical functions in vesicular transport [25]. Here we show that ARF1 is often abnormally overexpressed in breast cancer cells, and such overexpression is crucial to promote invasion and metastasis to be significantly associated with the poor outcomes of patients. Based on these facts, we demonstrate that ARF1 serves as a previously unidentified drug target and inactivating it can suppress metastatic breast cancer. The study of breast cancer metastasis is always hampered by a lack of reliable metastatic models. Although the importance of the *ARF1* gene has been determined using tail vein injection of immunocompromised SCID mice [26], intravascular injections only model extravasation and metastasis, and lack the growth characteristics and metastatic properties of human cancer [27]. Considering that NSG mice allow primary and metastatic tumors to develop coincidently [28], we established orthotopic breast cancer models in NSG mice to illustrate the contribution of *ARF1* alteration to cancer progression. Orthotopic implantation in mice indicates that loss of *ARF1* expression in breast cancer cells reduces risk of metastatic spread. These findings, together with the investigation in zebrafish, provide more solid evidence that ARF1 promotes breast cancer metastasis.

Mutations in *RAS* genes are very rare in human breast cancers, but RAS is pathologically hyperactivated in half of these cancers [29–31]. Since approaches to directly target RAS have not been successful, most efforts to block activated RAS have focused on pathways downstream, such as the MAPK and PI3K pathway [32, 33]. However, targeting these nodes in the signaling cascades individually typically involves a switch to the other pathway in a rescue strategy by the cancer cells to overcome monotherapies [34–37]. Previous studies demonstrate, similar to the oncogene RAS, ARF1 directly and activation-dependently activates the MAPK and PI3K pathway [7, 14, 26]. Therefore, inhibition of ARF1 may provide an alternative over the generally toxic simultaneous inhibition of multiple pathways by different drugs.

The exchange of GDP for GTP on ARFs is catalyzed by guanine nucleotide exchange factors (GEFs), and ARF- GEFs interact with ARFs through their Sec7 domain, which is necessary and sufficient for GEF activity [38–40]. Most existing ARF1 inhibitors block its activation by targeting the Sec7 domain on ARF1-GEFs [22]. Although inactivating a subset of ARF1-GEFs has been very useful for assessing the ARF1 function, ARF1 inhibitors remain a daunting challenge to develop into anti-cancer and anti-inflammatory drugs. For example, Brefeldin A (BFA), a known inhibitor stabilizing an abortive complex between the GEFs and ARF1 in the GDP-bound form, is highly cytotoxic [40]. Unlike BFA, Exo2 interferes with the function of ARF1 or ARF1-GEFs localized to the ER-Golgi intermediate compartment or the *trans-*Golgi network. While compared to BFA, Exo2 reduces ARF1 activation but the effect is much weaker [41]. Recently, a novel ARF1- ARFGEF inhibitor AMF-26, which differed structurally from BFA, has shown potential for inducing complete regression of human breast cancer BSY-1 xenografts *in vivo* [42]. However, no evidence has been provided that AMF26 and its derivatives has anti-metastasis effects. We have characterized a compound, LM11, specifically targets the ARF1-GDP/cytohesin-2 complex and acts as a non-competitive inhibitor [22]. In MDCK cells, LM11 suppresses ARNO-dependent migration because of its inhibition of ARF1 functions at the Golgi [22]. The data present here, for the first time, show potential therapeutic opportunities for breast cancer resulting from ARF1 inactivation in the context of LM11 treatment.

Recently, more general cancer characteristics such as genomic instability, invasiveness, and transplant ability apply to zebrafish tumors [19–21, 24, 43, 44]. As a model of whole organism, zebrafish can be used to facilitate better understanding of gene function involved in cancer progression and provide a means to develop promising preclinical agents. We evaluated ARF1 function and LM11 efficacy using the zebrafish-metastasis model. The value of fish assays is that the results can be obtained in an informative, cost-effective and time-efficient manner. The mice, therefore, will be used to confirm the effect of LM11 on suppression of metastasis.

## MATERIALS AND METHODS

### Cell lines and standard assays

Human breast cancer cell lines (MDA-MB-231, Hs578T and MCF7) and normal mammary epithelial cells MCF10A were directly obtained from American Type Culture Collection (ATCC, Rockville, MD). All the cell lines used in this study have been verified using SNP-CGH for characteristic cytogenetic changes or confirmed using STR DNA fingerprinting analysis [45, 46]. Standard cell culture, transient transfections, lentiviral transduction, western blot, real-time RT-PCR and cell proliferation assays were carried out as described previously [28, 45–47].

### Constructs, antibodies and other reagents

To stably knock down the *ARF1* gene, pLKO.1 lentiviral vectors harboring shRNA-targeting *ARF1* were obtained from Open Biosystems (Huntsville, AL). ARF1 and β-Actin antibodies were procured from Abcam (Cambridge, MA) and Sigma (St Louis, MO), respectively. The ARF1 inhibitor LM11 was obtained from ChemBridge (San Diego, CA). ARF1 activation was determined by the glutathione resin-bound GST-GGA3-PBD fusion protein as described previously [8].

### Cell viability and flow cytometric analysis

The cell viability was determined by CellTiter-Glo^®^ Luminescent cell viability assay (Promega, Madison, MI) and Zombie Aqua^TM^ fixable viability kit (BioLegend, San Diego, CA) according to manufacturer's instructions. Flow cytometry (LSR Fortessa cell analyzer, BD Biosciences, San Jose, CA) was used to record and analyze the cells stained with Zombie Aqua^TM^ dye with a maximum emission of 516 nm.

### Gap closure migration assays and invasion assays

Cell migration was performed using the Radius™ 24-well from Cell Biolabs (San Diego, CA). Briefly, cells were seeded on Radius cell migration plates and allowed to form monolayers. Circular gaps were generated by removing the gels and cells were treated with DMSO or LM11 for 24 hours. To compare differences in the migratory gap, phase-contrast images were captured at the same size using a Zeiss LSM-510 inverted microscope (Zeiss, Germany) and gap closure was analyzed using ImageJ. Cell migration velocity was calculated and statistically analyzed from three independent experiments. Cell invasion was performed using a Matrigel-coated modified Boyden chamber (BD biosciences, San Jose, CA) as described previously [45–47]. After incubation for 24 hours, cells on the upside were removed using cotton swabs, and the invading cells on the lower side were fixed and stained with 0.2% crystal violet. Numbers of the invading cells in six randomly selected fields were counted in each experiment using a Zeiss LSM-510 inverted microscope.

### Experimental metastasis assays

All experimental procedures were approved by the Institutional Animal Care and Use Committee (IACUC) at Augusta University. Tg(*kdrl:*EGFP) transgenic zebrafish which highlights the vasculature were maintained using established temperature and light cycle conditions as previously described [19, 48, 49]. In zebrafish metastasis assays, cancer cell transplantation was performed essentially as described previously [19]. Briefly, MDA-MB-231 cells infected with the lentivirus expressing control or *ARF1* shRNA were labeled with fluorescent dye CM-Dil (Life Technologies, Carlsbad, CA). Approximately 200 labeled MDA-MB-231 cells expressing *ARF1* shRNA or control shRNA were microinjected into the perivitelline space of 2 days post fertilization (dpf) Tg(*kdrl:*EGFP) embryos. The embryos were kept at 34°C and then imaged under anesthesia by confocal microscopy at 2 dpi. Percentage of metastasis was set as the number of embryos containing more than 5 cells outside the yolk sac. Total metastasis percentage was set as the total number of embryos with metastasis at 2 dpi relative to day zero. In mouse metastasis assays, *ARF1* knockdown MDA-MB-231 cells and the control cells were individually injected into 6-week-old female NSG mice (NOD.Cg- *Prkdc*^scid^*Il2rg*^tm1Wjl^/SzJ, Jackson Laboratory, Bar Harbor, ME) through the mammary fat pad under the fourth (abdominal) nipple as described previously [28]. Mice were sacrificed 6 weeks after injection and the lungs were then fixed in 10% neutral buffered formalin, embedded in paraffin blocks, sectioned at 5 μm, and subjected hematoxylin and eosin (H&E) staining.

### Zebrafish drug treatment

Tg(*kdrl:*EGFP) embryos at 2 dpf were injected with MDA-MB-231 cells and housed in 24-well plates containing a single larva per well in 500 μl filter-sterilized fish water. Fish were administrated with 0.5% DMSO or different doses of LM11 in the fish water at 4 hours post-injection (hpi). After 4 days of treatment, metastasis in fish body was analyzed using confocal microscopy. Percentage of metastasis was set as the number of embryos containing more than 5 cells outside the yolk sac. Total metastasis percentage was set as the total number of embryos with metastasis at 4 dpi relative to day zero.

### Tissue microarrays and IHC

The human breast tissue microarrays were purchased from US Biomax (Rockville, MD) and Novus Bio (Littleton, CO). IHC of the human tissue microarrays was conducted as described previously [7] using an ARF1 antibody (1:500). For quantifying staining intensity, 12 random microscopic fields were captured by a CCD camera (Olympus, Center Valley, PA) and signal intensity was determined using the Image-Pro Plus software (MediaCybernetics, Rockville, MD).

### Bioinformatics and statistical analysis

To determine the influence of *ARF1* expression on relapse-free survival of breast cancer patients, integrated available genome-level transcriptomic datasets from the Kaplan Meier (KM)-plotter [50] were assessed by stratifying patients based on the higher or lower *ARF1* expression. The genetic status of human *ARF* gene family related to cancer types was calculated from TCGA data by cBioPortal (http://www.cbioportal.org/) up to April 2016. The selected datasets in this study must contain a large number of patient samples (> 100). Expression data of *ARF1* in normal breast and breast cancer samples were obtained from Oncomine website (www.oncomine.org). Experiments shown are the means of multiple individual points from multiple experiments (± S.D.). A 2-tailed *P*-value of less than 0.05 was considered to indicate statistical significance.

## References

[1] Gupta GP, Massagué J (2006). Cancer metastasis: building a framework. Cell.

[2] Giuliano M, Giordano A, Jackson S, De Giorgi U, Mego M, Cohen EN, Gao H, Anfossi S, Handy BC, Ueno NT, Alvarez RH, De Placido S, Valero V (2014). Circulating tumor cells as early predictors of metastatic spread in breast cancer patients with limited metastatic dissemination. Breast Cancer Res.

[3] Chambers AF, Groom AC, MacDonald IC (2002). Dissemination and growth of cancer cells in metastatic sites. Nat Rev Cancer.

[4] Valastyan S, Weinberg RA (2011). Tumor metastasis: molecular insights and evolving paradigms. Cell.

[5] Nguyen DX, Massagué J (2007). Genetic determinants of cancer metastasis. Nat Rev Genet.

[6] Clark J, Moore L, Krasinskas A, Battey J, Tamkun J, Kahn RA (1993). Selective amplification of additional members of the ADP-ribosylation factor (ARF) family: cloning of additional human and Drosophila ARF-like genes. Proc Natl Acad Sci USA.

[7] Davis JE, Xie X, Guo J, Huang W, Chu WM, Huang S, Teng Y, Wu G (2016). ARF1 promotes prostate tumorigenesis via targeting oncogenic MAPK signaling. Oncotarget.

[8] Dong C, Li C, Wu G (2011). Regulation of α(2B)-adrenergic receptor-mediated extracellular signal-regulated kinase 1/2 (ERK1/2) activation by ADP-ribosylation factor 1. J Biol Chem.

[9] Bos JL, Rehmann H, Wittinghofer A (2007). GEFs and GAPs: critical elements in the control of small G proteins. Cell.

[10] Bernards A, Settleman J (2004). GAP control: regulating the regulators of small GTPases. J Cell Biol.

[11] Palacios F, Tushir JS, Fujita Y, D'Souza-Schorey C (2005). Lysosomal targeting of E-cadherin: a unique mechanism for the down-regulation of cell-cell adhesion during epithelial to mesenchymal transitions. Mol Biol Cell.

[12] Hashimoto A, Oikawa T, Hashimoto S, Sugino H, Yoshikawa A, Otsuka Y, Handa H, Onodera Y, Nam JM, Oneyama C, Okada M, Fukuda M, Sabe H (2016). P53-and mevalonate pathway-driven malignancies require Arf6 for metastasis and drugresistance. J Biol Chem.

[13] Cohen LA, Honda A, Varnai P, Brown FD, Balla T, Donaldson JG (2007). Active Arf6 recruits ARNO/cytohesin GEFs to the PM by binding their PH domains. Mol Biol Cell.

[14] Boulay PL, Cotton M, Melançon P, Claing A (2008). ADP-ribosylation factor 1 controls the activation of the phosphatidylinositol 3-kinase pathway to regulate epidermal growth factor-dependent growth and migration of breast cancer cells. The J Biol Chem.

[15] Boulay PL, Schlienger S, Lewis-Saravalli S, Vitale N, Ferbeyre G, Claing A (2011). ARF1 controls proliferation of breast cancer cells by regulating the retinoblastoma protein. Oncogene.

[16] Lewis-Saravalli S, Campbell S, Claing A (2013). ARF1 controls Rac1 signaling to regulate migration of MDA-MB-231 invasive breast cancer cells. Cell Signal.

[17] Schlienger S, Ramirez RA, Claing A (2015). ARF1 regulates adhesion of MDA-MB-231 invasive breast cancer cells through formation of focal adhesions. Cell Signal.

[18] Haines E, Schlienger S, Claing A (2015). The small GTPase ADP-Ribosylation Factor 1 mediates the sensitivity of triple negative breast cancer cells to EGFR tyrosine kinase inhibitors. Cancer Biol Ther.

[19] Teng Y, Xie X, Walker S, White DT, Mumm JS, Cowell JK (2013). Evaluating human cancer cell metastasis in zebrafish. BMC Cancer.

[20] Shao J, Teng Y, Padia R, Hong S, Noh H, Xie X, Mumm JS, Dong Z, Ding HF, Cowell J, Kim J, Han J, Huang S (2013). COP1 and GSK3β cooperate to promote c-Jun degradation and inhibit breast cancer cell tumorigenesis. Neoplasia.

[21] Hong S, Noh H, Teng Y, Shao J, Rehmani H, Ding HF, Dong Z, Su SB, Shi H, Kim J, Huang S (2014). SHOX2 is a direct miR- 375 target and a novel epithelial-to-mesenchymal transition inducer in breast cancer cells. Neoplasia.

[22] Viaud J, Zeghouf M, Barelli H, Zeeh JC, Padilla A, Guibert B, Chardin P, Royer CA, Cherfils J, Chavanieu A (2007). Structure-based discovery of an inhibitor of Arf activation by Sec7 domains through targeting of protein-protein complexes. Proc Natl Acad Sci USA.

[23] Flisiak S, Zeeh JC, Guibert B, Cherfils J, Zeghouf M (2008). An Arf1 GTPase mutant with different responses to GEF inhibitors. Biochemical and Biochem Biophys Res Commun.

[24] Xie X, Ross JL, Cowell JK, Teng Y (2015). The promise of zebrafish as a chemical screening tool in cancer therapy. Future Med Chem.

[25] Donaldson JG, Jackson CL (2011). ARF family G proteins and their regulators: roles in membrane transport, development and disease. Nat Rev Mol Cell Biol.

[26] Schlienger S, Campbell S, Pasquin S, Gaboury L, Claing A (2016). ADP-ribosylation factor 1 expression regulates epithelial-mesenchymal transition and predicts poor clinical outcome in triple-negative breast cancer. Oncotarget.

[27] Simmons JK, Hildreth BE, Supsavhad W, Elshafae SM, Hassan BB, Dirksen WP, Toribio RE, Rosol TJ (2015). Animal Models of Bone Metastasis. Vet Pathol.

[28] Teng Y, Ren X, Li H, Shull A, Kim J, Cowell JK (2016). Mitochondrial ATAD3A combines with GRP78 to regulate the WASF3 metastasis-promoting protein. Oncogene.

[29] Eckert LB, Repasky GA, Ulkü AS, McFall A, Zhou H, Sartor CI, Der CJ (2004). Involvement of Ras activation in human breast cancer cell signaling, invasion, and anoikis. Cancer Res.

[30] von Lintig FC, Dreilinger AD, Varki NM, Wallace AM, Casteel DE, Boss GR (2000). Ras activation in human breast cancer. Breast Cancer Res Treat.

[31] Baines AT, Xu D, Der CJ (2011). Inhibition of Ras for cancer treatment: the search continues. Future Med Chem.

[32] Gysin S, Salt M, Young A, McCormick F (2011). Therapeutic strategies for targeting ras proteins. Genes Cancer.

[33] Carracedo A, Ma L, Teruya-Feldstein, Rojo F, Salmena L, Alimonti A, Egia A, Sasaki AT, Thomas G, Kozma SC, Papa A, Nardella C, Cantley LC (2008). Inhibition of mTORC1 leads to MAPK pathway activation through a PI3K-dependent feedback loop in human cancer. J Clin Invest.

[34] Ebi H, Corcoran RB, Singh A, Chen Z, Song Y, Lifshits E, Ryan DP, Meyerhardt JA, Benes C, Settleman J, Wong KK, Cantley LC, Engelman JA (2011). Receptor tyrosine kinases exert dominant control over PI3K signaling in human KRAS mutant colorectal cancers. J Clin Invest.

[35] Floc'h N, Kinkade CW, Kobayashi T, Aytes A, Lefebvre C, Mitrofanova A, Cardiff RD, Califano A, Shen MM, Abate-Shen C (2012). Dual targeting of the Akt/mTOR signaling pathway inhibits castration-resistant prostate cancer in a genetically engineered mouse model. Cancer Res.

[36] Turke AB, Song Y, Costa C, Cook R, Arteaga CL, Asara JM, Engelman JA (2012). MEK inhibition leads to PI3K/AKT activation by relieving a negative feedback on ERBB receptors. Cancer Res.

[37] Roberts PJ, Usary JE, Darr DB, Dillon PM, Pfefferle AD, Whittle MC, Duncan JS, Johnson SM, Combest AJ, Jin J, Zamboni WC, Johnson GL, Perou CM (2012). Combined PI3K/mTOR and MEK inhibition provides broad antitumor activity in faithful murine cancer models. Clin Cancer Res.

[38] Shin HW, Nakayama K (2004). Guanine nucleotide-exchange factors for arf GTPases: their diverse functions in membrane traffic. J Biochem.

[39] Cherfils J, Ménétrey J, Mathieu M, Le Bras G, Robineau S, Béraud-Dufour S, Antonny B, Chardin P (1998). Structure of the Sec7 domain of the Arf exchange factor ARNO. Nature.

[40] Honda A, Al-Awar OS, Hay JC, Donaldson JG (2005). Targeting of Arf-1 to the early Golgi by membrin, an ER-Golgi SNARE. J Cell Biol.

[41] Bourgoin SG, El Azreq MA (2012). Small inhibitors of ADP-ribosylation factor activation and function in mammalian cells. World J Pharmacol.

[42] Ohashi Y, Iijima H, Yamaotsu N, Yamazaki K, Sato S, Okamura M, Sugimoto K, Dan S, Hirono S, Yamori T (2012). AMF-26, a novel inhibitor of the Golgi system, targeting ADP-ribosylation factor 1 (Arf1) with potential for cancer therapy. J Biol Chem.

[43] Feitsma H, Cuppen E (2008). Zebrafish as a cancer model. Mol Cancer Res.

[44] Patton EE (2012). Live imaging in zebrafish reveals neu(trophil) insight into the metastatic niche. J Pathol.

[45] Teng Y, Mei Y, Hawthorn LA, Cowell JK (2014). WASF3 regulates miR-200 inactivation by ZEB1 through suppression of KISS1 leading to increased invasiveness in breast cancer cells. Oncogene.

[46] Teng Y, Bahassan A, Dong D, Hanold LE, Ren X, Kennedy EJ, Cowell JK (2016). Targeting the WASF3-CYFIP1 complex using stapled peptides suppresses cancer cell invasion. Cancer Res.

[47] Teng Y, Pi W, Wang Y, Cowell JK (2016). WASF3 provides the conduit to facilitate invasion and metastasis in breast cancer cells through HER2/HER3 signaling. Oncogene.

[48] Teng Y, Xie X, Walker S, Rempala G, Kozlowski DJ, Mumm JS, Cowell JK (2010). Knockdown of zebrafish Lgi1a results in abnormal development, brain defects and a seizure-like behavioral phenotype. Hum Mol Genet.

[49] Teng Y, Xie X, Walker S, Saxena M, Kozlowski DJ, Mumm JS, Cowell JK (2011). Loss of zebrafish lgi1b leads to hydrocephalus and sensitization to pentylenetetrazol induced seizure-like behavior. PLoS One.

[50] Györffy B, Lanczky A, Eklund AC, Denkert C, Budczies J, Li Q, Szallasi Z (2010). An online survival analysis tool to rapidly assess the effect of 22,277 genes on breast cancer prognosis using microarray data of 1,809 patients. Breast Cancer Res Treat.

